# Management and long-term evolution of a patient with 3-hydroxy-3-methylglutaryl-coenzyme A lyase deficiency

**DOI:** 10.1186/s13052-017-0333-4

**Published:** 2017-01-19

**Authors:** Juan Ignacio Muñoz-Bonet, María del Carmen Ortega-Sánchez, José Luis León Guijarro

**Affiliations:** 1 Pediatric Intensive Care Unit, Hospital Clínico Universitario, University of Valencia, Av. Blasco Ibáñez 17, 46010 Valencia, Spain; 2 Department of Pediatrics, Hospital Clínico Universitario, Av. Blasco Ibáñez 17, 46010 Valencia, Spain; 3 Department of Radiology, Hospital Clínico Universitario, Avda. Blasco Ibáñez 17, 46010 Valencia, Spain

**Keywords:** HMG-CoA lyase deficiency, Reye-like syndrome, Alcohol consumption, Hyperamoniemia, Sodium phenylacetate and sodium benzoate, Cytotoxic brain edema, Cerebral vasospasm

## Abstract

**Background:**

3-Hydroxy-3-methylglutaryl-coenzyme A (HMG-CoA) lyase deficiency is a rare inborn error of metabolism characterized by recurrent metabolic crises caused by fasting, intercurrent illness and excessive physical exercise. Non ketotic hypoglycemia is normally the cause of primary symptoms but without an immediate treatment the illness can evolve into a worsening metabolic state resembling the Reye’s syndrome that may cause the patient’s death. We report a case with some clinical and therapeutic features not previously described.

**Case presentation:**

Patient with HMG-CoA lyase deficiency whom after diagnosis at 2 years of age was re-admitted 12 years later, after severe metabolic decompensation following consumption of alcohol. Despite a quick correction of hypoglycemia, within the following few hours, the patient fell into a coma. Suspecting intracranial hypertension (ICH), the patient required mechanical ventilation. Although liver cytolysis was minimal, hyperamoniemia reached 1394 μmol/L, returning to normal, a few hours after administering sodium phenylacetate and sodium benzoate, whose use has not been reported in these patients. Brain edema was evidenced in the computed tomography and by the magnetic resonance imaging that determined that the edema was cytotoxic, as quantified with the restriction of diffusion in the apparent diffusion coefficient map. During the recovery of the ICH, we belatedly, detected vasospasm moderate-severe that was treated with nimodipine. Currently, the patient maintains clinical normality.

**Conclusions:**

The alcohol consumption must be avoided in patients with HMG-CoA lyase deficiency. In our patient hyperamoniemia was effectively treated with sodium phenylacetate and sodium benzoate. Magnetic resonance imaging showed and quantified the cytotoxic brain edema. Belatedly, a cerebral vasospasm was an additional mechanism of cerebral injury. None of these observations has been previously reported.

## Background

3-Hydroxy-3-methylglutaryl-coenzyme A (HMG-CoA) lyase deficiency is a recessive autosomal hereditary disease described for the first time by Faull et al. [1]. This mitochondrial enzyme catalyzes the cleavage of HMG-CoA to form acetyl-CoA and acetoacetate, which is the common final step of ketogenesis and leucine degradation [2]. The illness is characterized by recurrent metabolic crises which generally start in the early neonatal period (30%) or in the first year of life (60%) [3]. These crises are caused by fasting, intercurrent illness and excessive physical exercise [2].

The non ketotic hypoglycemia is normally the cause of primary symtoms. But the illness can evolve into a worsening metabolic state resembling the Reye’s syndrome [3]. Such crises can cause death in 20% of cases and serious long term health predicaments such as psychomotor retardation, epilepsy, hepatic steatosis, pancreatitis or dilated cardiomyopathy [2].

Patients with HMG-CoA lyase deficiency show a diagnostic urinary organic acids pattern [1, 4]. Confirmation diagnosis requires determining the enzyme activity or a genetic study. The altered gene is the HMGCL, located on chromosome 1p36.1 and 48 mutations have been identified [5, 6]. The most common in Mediterranean countries and the second most common in the world is the E37X [7, 8].

We report a new case of HMG-CoA lyase deficiency whom after the diagnosis at 2 years of age was once again re-admitted 12 years later, after severe metabolic decompensation following consumption of alcohol.

## Case presentation

Following 12 h of food rejection and vomiting, due to a cold, our 2 years old Spanish child was admitted into the Pediatric Intensive Care Unit (PICU) with clinical symptoms of hypoactivity, hypotonia, vomiting and transient loss of consciousness. The patient had no previous illness of interest. The patient’s parents were Moroccan, consanguineous and without any illness of note.

Patient examination on admission showed a Glasgow Coma Scale (GCS) of 13–14, tiredness, hypotonia and mild hepatomegaly. Vital signs were all normal. Blood analysis highlighted severe hypoglycemia (29 mg/dL) without ketosis, moderate metabolic acidosis and hyperlactatemia (10.3 mmol/L). The ammonium level was slightly elevated (86.7 μmol/L). Following the intravenous administration of glucose there was an immediate and sustained stabilization of blood glucose. The following 12 h also saw the normalization of metabolic acidosis, hyperlactatemia and consciousness. An electroencephalogram (EEG) showed no significant pathological alterations and the magnetic resonance imaging (MRI) indicated small hyperintensity in FLAIR and T_2_ sequences in the frontal subcortical white matter suggestive of demyelination areas. The metabolic study showed increased urinary metabolites of leucine, suspecting HMG-CoA lyase deficiency. The diagnostic confirmation was done via a genetic study: the patient was homozygous for the E37X mutation. The patient received treatment with L-carnitine and outpatient control until 3 years of age when he cut short his attendance.

At 14 years of age, he returned to our Pediatric Emergency Department due to a loss of consciousness following vomiting brought on by alcohol consumption a few hours before (3 vodkas with coke). The family made no reference to previous illnesses or admissions, excepting some infant resolved episodes of hypoglycemia. On examination the patient was unconscious, with ocular revulsion, generalized tremors and hypertonia, suggestive of generalized tonic seizure. All other vital signs were as normal. A blood analysis highlighted severe hypoglycemia (8 mg/dL) without ketosis, metabolic acidosis (pH 7.28, pC0_2_ 32.4 mmHg, HC0_3_^−^ 14.9 mEq/L, base excess −10.4 mEq/L) and hyperlactatemia (5.6 mmol/L). The biochemical blood evolution is shown in Table 1. A quick correction of hypoglycemia was performed with intravenous administration of glucose, which led to an initial recovery of the patient’s awareness (GCS 13–14).

**Table 1:** Blood biochemistry evolution. N-Carbamil glutamate (100 mg/kg through nasogastric tube) was administered at 2 h after PICU admission, and Sodium phenylacetate and sodium benzoate (Ammonul® 55 mL/m^2^) was administered after 8 h. A rapid increase in hyperammonemia may be observed, peaking at 7 h after admission to the PICU and a subsequent rapid decline to normalization between 14 and 19 h. The liver and Kidney function improved from 24 h of hospital admission. AST, aspartate aminotransferase; ALT, alanine aminotransferase; PT, Prothrombin Time; QI, Quick index; APTT (sec) activated partial thromboplastin time; CK, creatine phosphokinase; CRP, C-reactive protein

PICU Admission		6 h	7 h	11 h	14 h	19 h
Ammonium (μmol/L)	669	1075	1394	522	70,6	24,4
Hospital Admission		12 h	24 h	48 h	72 h	5° day
Urea (mg/dL)	39	48	102	69	53	35
Creatinine (mg/dL)	0.92	1.25	1.31	0.94	0.59	0.41
Uric acid (mg/dL)	-	-	-	13.4	11.1	5.9
AST/ALT (U/L)	74/42	172/92	157/104	82/82	42/58	38/54
Total Bilirubin (mg/dL)	1.83	2.2	0.86	0.83	0.65	0.37
PT (sec) / QI (%)	19.8/51	25.8/35	26.7/32	16.3/65	11.1/100	-
APTT (sec)	30.2	31	37.5	37.3	30	-
Factor VII (%)	-	-	16.8	45.2	-	-
D-dimer (ng/mL)	-	161	-	410	457	-
CK (U/L)	285	-	718	257	132	-
CRP (mg/L)	2.4	2.4	2.2	65.7	121.4	16

Despite maintaining normal glucose levels, the following 4 h witnessed further neurological deterioration, alternating between phases of agitation and progressive lethargy. Suspecting intracranial hypertension (ICH), the patient was admitted to the PICU. Treatment was initiated with anti-edema measures (mannitol, furosemide and postural treatment), and intravenous administration of L-carnitine (100 mg/kg). After the first measurement of ammonium, carglumic acid was administered through a nasogastric tube. An urgent cranial computed tomography (CT) scan was performed, which detected chronic defects in white matter but without signs of cerebral edema. In the 8 h after PICU admission there was a continued deterioration to GCS < 9. The patient required invasive mechanical ventilation. In order to treat the hyperammonaemia sodium phenylacetate and sodium benzoate was administered intravenously and although continuous venovenous haemodiafiltration was prepared, it was not used due to a rapid decline in hyperammonaemia until complete normalization within a few hours (Table 1).

Using bispectral index monitoring (BIS), electrical brain activity registered values between 25–40. At 24 h the EEG showed severe and generalised signs of slowdown in cerebral bioelectrical activity, transcranial Doppler (TCD) a diffuse slight-moderate increase of the pulsatility index (1.1–1.3), compatible with mild ICH, and CT scan showed a diffuse worsening in the cortical-subcortical differentiation and a decreased size of ventricles and subarachnoid space. All consistent with cerebral edema.

**Fig. 1:**
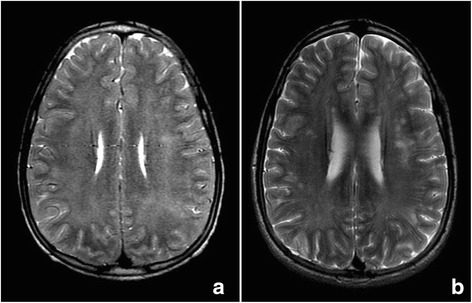
Axial section MRI T_2_-weighted sequences at 72 h. Image **a** indicates an increased intensity in white matter of diffuse bilateral distribution. Image **b** taken after 8 months; indicates resolution of the previous diffuse affectation, however the appearance of specific areas with increased signal in the periventricular brain parenchyma predominantly of the left side, indicative of definitive residual injury

**Fig. 2:**
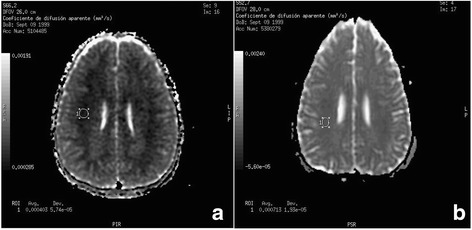
Diffusion-Weighted Imaging in MRI, comparing the ADC map, at 72 h (Image **a**) and after 8 months (Image **b**). It may be observed that the circular region of interest to determine the ADC value in both images: with values of: 0,4 × 10^−3^ mm^2^/s at 72 h (restricted diffusion), whilst after eight months the value was 0.7 × 10^−3^ mm^2^/s (normal ADC map value)

At 48 h, the EEG detected a presence of slow waves but with remarkable bioelectrical improvement, by which sedation was withdrawn. The TCD showed an increase in average speeds (180–190 cm/s) in all the territories with predominance in the left hemisphere, compatible with moderate-severe vasospasm. Thus treatment was started with intravenous nimodipine. The patient maintained BIS values <50 in the following 12 h, later waking up and being subsequently extubated after 72 h of PICU admission (GCS 13–14). The subsequent MRI showed hyperintensity white matter signals in FLAIR and T_2_ sequences, in relation to base metabolic leucoencephalopathy (Fig. 1a), but also indicated new sources which in the diffusion-weighted imaging, showed a reduced apparent diffusion coefficient (ADC) indicative of cytotoxic edema (Fig. 2a).

On the 8^th^ day neurological examination was normal as that of EEG and TCD testing, withdrawing treatment with nimodipine. Once again, when the patient was discharged we recommended continued treatment with L-carnitine. The MRI performed at 8 months indicated a normalization of ADC, but with the presence of specific areas with increased signal in T_2_ and FLAIR sequences indicative of definitive residual injury in the periventricular brain parenchyma as well as those areas of previous demyelination (Figs. 1b and 2b). Currently, the patient maintains clinical normality.

## Discussion

In patients with HMG-CoA lyase deficiency the main mechanism that produces fasting hypoglycemia is an excess of glucose use by the peripheral tissues, since these cannot have ketones nor free fatty acids for oxidation, due to the shortfall of secondary carnitine. This deficit is the result of the excess of acyl-CoA, which after esterification to acyl-carnitine, is lost in the urine [2, 4, 6]. For these reasons, these patients should maintain dietary supplementation with L-carnitine (75–100 mg/kg/day) [9]. Our patient did not follow this recommendation at the age of 14, probably because the patient remained asymptomatic for several years and the family lost consciousness of the chronic nature of this illness. It is possible that this deficit facilitates the development of Reye’s syndrome, as has been described in other enzymatic defects of fatty acid oxidation. In addition, in this episode the consumption of alcohol could aggravate the metabolic crisis by blocking the neoglucogenesis from pyruvate.

As mentioned, the illness may evolve to a Reye syndrome and has been reported to cause severe cytolysis. However, in our patient only a minimum elevation of transaminases occurred. Therefore, the absence of hepatic cytolysis does not preclude the existence of a serious hepatic encephalopathy. For these reasons, the blood levels of ammonium must be determined in any decompensation. In our patient intravenous benzoate and sodium phenyl acetate has been a very effective treatment of hyperammonaemia. It has been suggested that this treatment could be useful in these patients [10] but has not been reported previously. The evolution of Quick index and Factor VII suggest that the liver synthesis function was also affected. The normal values of D-dimer support this hypothesis. The minimal cytolysis could also explain the rapid normalization of liver function and the recovery of the patient with detox and supportive treatment in PICU.

Characteristically, the neuroimaging studies carried out on these patients show diffuse cerebral white matter hypodensity on CT scan and hyperintensity on T_2_ weighted MRI studies [3, 5]. However, there is less information on the findings of the neuroimaging tests during an acute episode. In the MRI, we detected that the cerebral edema was of the cytotoxic type with the restriction of diffusion in the ADC map. In it, the swelling cell produces a decrease in the extracellular space and a decrease in the molecular diffusion that is quantified and that in our patient was diminished in the initial study, normalizing in the test carried out 8 months later. This finding has not been previously described.

Cerebral infarction in these patients has been related with biochemical mechanism and not with loss of blood supply [11]. However, in addition to the ICH, in our patient the TCD performed at 48 h detected another vascular mechanism of brain injury not previously described. As such the vasospasm could have belatedly aggravated cerebral ischemic damage after the improvement of the ICH. Perhaps this mechanism could explain the ischemic cerebral infarction with vascular distribution described by Huemer et al. that occurred 4 days after metabolic decompensation [12]. Despite the apparent full clinical recovery of our patient, it should be noted that the MRI performed after 8 months detected residual injuries in the periventricular area of cerebral parenchyma of left dominance, where the vasoespasm detected by TCD was greater. For this reasons we think that TCD monitoring must be used in this patients.

It has been reported by several authors that with age there is an increased tolerance to fasting [2, 10]. However, we want to point out that this genetic disease does not heal. Thus, in our patient the most serious metabolic crisis occurred 12 years after diagnosis, when the family had already lost conscientious awareness of the disease. For these reasons, the family must be made aware of the chronic nature of the disease.

## Conclusions

The alcohol consumption must be avoided in patients with HMG-CoA lyase deficiency because it can precipitate and exacerbate the Reye-like syndrome episodes. The absence of hepatic cytolysis does not preclude the existence of a serious hepatic encephalopathy. In our patient hyperamoniemia was effectively treated with sodium phenylacetate and sodium benzoate. In the acute episode MRI showed and quantified the cytotoxic brain edema. Belatedly, a cerebral vasospasm was an additional mechanism of cerebral injury. Finally this patients must avoid fasting and maintain L-carnitine supplementation.

## Abbreviations

ADC: Apparent diffusion coefficient
BIS: Bispectral index
CT: Computed tomography
EEG: Electroencephalogram
FLAIR: Fluid-attenuated inversion recovery
GCS: Glasgow coma scale
HMG-CoA: 3-Hydroxy-3-methylglutaryl-coenzyme A
ICH: Intracranial hypertension
MRI: Magnetic resonance imaging
PICU: Pediatric intensive care unit
TCD: Transcranial doppler

## References

[1] Faull K, Bolton P, Halpern B (1976). Patient with defect in leucine catabolism. N Engl J Med.

[2] Santarelli F, Cassanello M, Enea A (2013). A neonatal case of 3-hydroxy-3-methylglutaric-coenzyme A lyase deficiency. Ital J Pediatr.

[3] Zafeiriou DI, Vargiami E, Mayapetek E, Augoustidou-Savvopoulou P, Mitchell GA (2007). 3-Hydroxy-3-methylglutaryl coenzyme A lyase deficiency with reversible white matter changes after treatment. Pediatr Neurol.

[4] Dos Santos MM, Ribas GS, Wayhs CA (2015). Increased oxidative stress in patients with 3-hydroxy-3-methylglutaric aciduria. Mol Cell Biochem.

[5] Reimao S, Morgado C, Almeida IT, Silva M, Real HC, Campos J (2009). 3-Hydroxy-3-methylglutaryl-coenzyme A lyase deficiency: Initial presentation in a young adult. J Inherit Metab Dis.

[6] Leipnitz G, Regla Vargas C, Wajener M (2015). Disturbance of redox homeostasis as a contributing underlying pathomechanism of brain and liver alterations in 3-hydroxy-3-methylglutaryl-CoA lyase deficiency. J Inherit Metab Dis.

[7] Pié J, Casals N, Casale CH (1997). A nonsense mutation in the 3-hydroxy-3-methylglutaryl-CoA lyase gene produces exon skipping in two patients of different origin with 3-hidroxy-3-methylglutaryl-CoA lyase deficiency. Biochem J.

[8] Mir C, Lopez-Viñas E, Aledo R (2006). A single-residue mutation, G203E, causes 3-hydroxy-3-methylglutaric aciduria by occluding the substrate channel in the 3D structural model of HMG-CoA lyase. J Inherit Metab Dis.

[9] Pierron S, Giudicelli H, Moreigne M (2010). Déficit en 3-HMG-CoA lyase à révélation tardive: savoir reconnaitre une maladie rare mais traitable. Arch Pediatr.

[10] Gibson KM, Breuer J, Nyhan WL (1988). 3-Hydroxy-3-methylglutaryl-coenzyme A lyase deficiency: review of 18 reported patients. Eur J Pediatr.

[11] Muroi J, Yorifuji JT, Uematsu A, Nakajata T (2000). Cerebral infarction and pancreatitis: possible complications of patients with 3-hydroxy-3-methylglutaryl-CoA lyase deficiency. J Inherit Metab Dis.

[12] Huemer M, Muehl A, Wandl-Vergesslich K, Strobl W, Wanders RJ, Stoeckler-Ipsiroglu S (1998). Stroke-like encephalopathy in an infant with 3-hydroxy-3-methylglutaryl-coenzyme A lyase deficiency. Eur J Pediatr.

